# Fellow-Workers and the Roll of Honour

**Published:** 1915-05-29

**Authors:** 


### ROUND THE WORLD.

[The Editor invites Early Information and Contributions Marked "Fellow-Workers."]

### Fellow-Workers and the Roll of Honour.

The sad list of those who have died whilst prisoners of war in a foreign country includes that of Captain Stephen Field, R.A.M.C., who succumbed at the Wittenburg Camp in Saxony on April 10. Educated at St. Mary's Hospital, where for many years his father, the late Mr. George P. Field, held a prominent position as aural surgeon and dean of the medical school, this ill-fated officer qualified L.R.C.P., M.R.C.S. in 1906, and after serving as assistant house surgeon at the Coventry and Warwick Hospital was successful in obtaining a commission in the Army Medical Service.

Further, the deaths of three other young R.A.M.C. officers whilst still in their twenties have to be recorded this week, these medical heroes being Lieutenants G. H. Lunan, J. A. McMahon, and M. Pern.

Lieutenant George Harold Lunan graduated in medicine and surgery at Edinburgh two years ago. The son of a pharmaceutical chemist in that city, Mr. George Lunan, he went to Daniel Stewart's College in earlier years. Last October he was appointed to the 9th Lancers, with which regiment he served until killed at the post of duty on May 13, his brief career terminating at the age of twenty-three years. Only a few days previously he had been congratulated on a remarkable escape from death.

Lieutenant John Aquila McMahon recently came back wounded as a result of the Ypres struggle, but was unable to rally his strength, and died on May 12. Born in 1890 at Howth, in Ireland, the late officer graduated M.B., B.Ch. at Dublin in 1913. Taking a temporary commission in the R.A.M.C. in September, he subsequently served with the Somerset Light Infantry.

Lieutenant Montague Pern, R.A.M.C., fell at Ypres on May 9. In his twenty-seventh year, he had been qualified three years, having passed his student days at Guy's Hospital. When war broke out he was assistant medical officer at the Portsmouth Workhouse Infirmary, which post he left almost immediately to join the Army. Lieutenant Pern's father was the late Mr. Alfred Pern, F.R.C.S., of Botley.

Of the three medical officers carried by the unfortunate battleship *Goliath*, Fleet-Surgeon Waters was apparently the only one to lose his life on that terrible night of disaster in the Dardanelles. A Queen's College, Galway, student, Mr. George Alexander Waters became an M.D., M.Ch. in 1884, and entered the Navy soon after qualification. Attaining the rank of Fleet-Surgeon in 1903 he was becoming one of the more senior officers of his service when appointed to the *Goliath* last August.

The *Medical Officer* publishes the names of several more members of the public health services who have lately joined the Royal Army Medical Corps as temporary medical officers. These include Mr. T. R. Bowen, Swansea; Dr. G. F. Buchan, Willesden; W. F. Corfield, Colchester; J. Dawson, Wigtown; A. T. Nankivell, Poole; F. T. C. Linton, Tunbridge Wells; A. S. Parkinson, Staffordshire; W. S. Stalker, Swinton; and A. White, Staffordshire.

According to the same journal Dr. Thomas Evans, medical officer of health for Swansea, finds it impossible to get away for military service as he would have liked owing to the important nature of his civil work and the fact that Mr. Thomas Rufus Bowen (a former Cardiff and London Hospital student), his assistant, has already joined the Army. The Swansea Town Council have felt that it would be unwise to place the responsible duties of medical officer of health to such a large centre of population and port completely in strange hands.

Our contemporary also announces that Dr. W. A. Wetwan, medical officer of health for the Bridlington Rural District, has been posted to the Northumbrian casualty clearing station, where he will retain the rank of major held by him in the 5th Battalion of the Yorkshire Regiment (Alexandra Princess of Wales' Own).

Dr. George Frederick Buchan, M.D. Glasg., is the present medical officer of health and school medical officer for Willesden, to which he was appointed after serving in a similar capacity for Heston and Isleworth. At Glasgow, where he graduated M.B., Ch.B. with distinctions in 1906, Dr. Buchan won the Alexander Donaldson scholarship and J. A. Patterson bursary; then, taking the D.P.H. Camb. soon after qualifying, he devoted himself to State medicine, and served an apprenticeship as assistant medical officer of health in Birmingham and Essex.

Dr. Walter Francis Corfield temporarily leaves the combined posts of medical officer of health, school medical officer, and public analyst for Colchester now that he is doing military work. Qualifying at the Conjoint Board's examinations from University College Hospital in 1904, he subsequently became house physician there, and reading for the London degree took the M.B., B.S. in 1907 and the M.D. in State medicine four years later. For a time Dr. Corfield acted as assistant in the bacteriological laboratory at University College Hospital Medical School.

Dr. James Dawson, who relinquishes control of the public health work at Wigtown and adjacent districts for the time being, had considerable experience of work in the field at the time of the South African War. Going to the Front as civil surgeon, he spent some two and a half years in South Africa, during which period he officiated as analyst to the Cape Town base and as officer in charge of an ambulance train. He became an M.B., C.M. of Aberdeen in 1894, six years later taking the public health diploma with honours.

Dr. Frederick Thomas Churchill Linton, released from work at Tunbridge Wells for the time being, has been responsible for the sanitary service, school inspection, and isolation hospital there, in addition to which he has been police surgeon for the district. An Edinburgh man, he qualified M.B., Ch.B. twelve years ago, and had much experience of State medicine as assistant at Leith, Croydon, and Norwich before becoming a full-blown medical officer of health.

Dr. Adam White, M.B., Ch.B. Edin., D.P.H. Cantab., leaves his work as chief tuberculosis officer for North Staffordshire on joining the R.A.M.C. A former student of both Edinburgh and Manchester, he has held the appointments of resident house surgeon at Edinburgh Royal Infirmary, resident medical officer to Sheffield City Hospital, and tuberculosis officer for Hereford.

